# Spectral Entropy Analysis and Source-Level EMI Suppression in Inverters via Sequential Switching of Series-Connected IGBTs

**DOI:** 10.3390/e28060665

**Published:** 2026-06-10

**Authors:** Shuo Gao, Xu Wang

**Affiliations:** The College of Information Science and Engineering, Northeastern University, Shenyang 110819, China; wangxu@ise.neu.edu.cn

**Keywords:** spectral entropy, EMI suppression, voltage shaping, series-connected IGBTs, power electronics complexity

## Abstract

This paper proposes a source-level electromagnetic interference suppression strategy for high-voltage inverters that uses a series-connected IGBT topology and discrete staircase voltage shaping. From an information-theoretic perspective, the staircase shaping transforms chaotic wideband switching noise into a deterministic harmonic structure, thereby reducing the spectral entropy of the EMI source. This information optimization is achieved using a CPLD-based sequential gate drive circuit, which eliminates the need for complex active gate profiling algorithms. Experimental results obtained using a 1140 V explosion-proof motor drive platform demonstrate harmonic attenuation of 4–16 dB μV within a 2 MHz band. Importantly, this targeted entropy reduction occurs alongside a 68.7% reduction in active-region switching losses, suggesting a concurrent decrease in local thermodynamic entropy production during switching transients. Increasing spectral determinism and relaxing requirements for subsequent physical filters effectively lower the conditional entropy of the overall electromagnetic environment. Leveraging the structural flexibility of series IGBTs, this method provides a practical, low-complexity solution and establishes a novel framework between power electronics and information theory for electromagnetic compatibility.

## 1. Introduction

**Figure 1:**
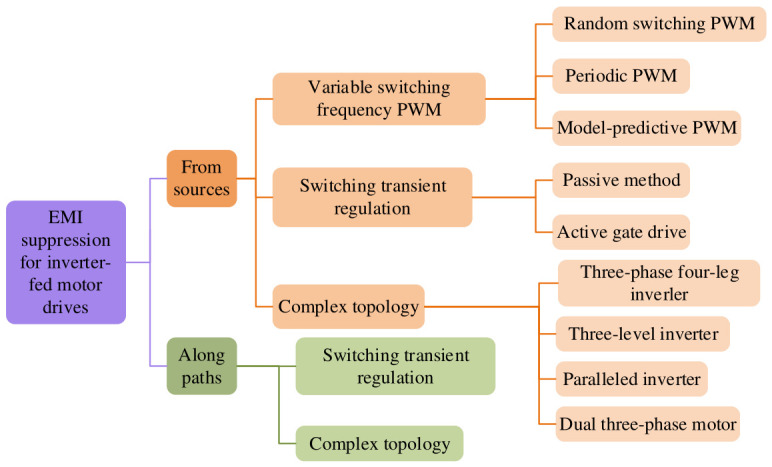
Categorization of EMI suppression for inverter-fed motor drives.

Inverter-fed motor drive systems are critical to the power conversion backbone of medium- and high-voltage industrial applications. Due to their technological maturity, cost-effectiveness and operational reliability, insulated-gate bipolar transistors (IGBTs) remain the predominant choice of switching device in these architectures [1,2]. However, the fundamental mechanism of pulse width modulation (PWM) dictates that rapid IGBT switching transitions generate discrete, sharp voltage edges. These aggressive energetic transitions manifest as electromagnetic interference (EMI), characterized by severe spectral complexity and chaotic high-frequency noise [3,4]. If left unmanaged, this disordered electromagnetic environment can cause cascading system failures ranging from accelerated bearing degradation to corruption of critical analogue feedback signals, ultimately endangering the operational integrity of the drive. Consequently, EMI suppression strategies spanning generation mitigation at the source [5] to attenuation along propagation paths [6], as shown in Figure 1, must be integrated into the system design. As conventional passive filters inevitably increase the cost, volume and weight of the system, regulating the informational and energetic intensity of EMI directly at the source has become a key area of research.

Although multi-level and parallel inverter topologies mitigate source-side electromagnetic interference (EMI) by reducing voltage steps, they increase hardware costs and architectural complexity. This restricts their use to specific high-power applications rather than general EMI suppression [7,8]. Consequently, mainstream research has shifted toward variable-switching-frequency pulse-width modulation (VSFPWM) and switching transient regulation. VSFPWM techniques, including random [8,9,10], periodic [11,12], and model-predictive PWM [13], operate by dynamically altering the pulse distribution. Numerous refinements have been proposed to optimize VSFPWM’s spread-spectrum performance and current ripple control [14,15,16,17,18,19]. From an information-theoretic perspective, however, VSFPWM relies on frequency dithering to redistribute localized harmonic energy across a much broader spectral band. Rather than eliminating electromagnetic disturbance, these methods actively “whiten” the noise, artificially maximizing the spectral entropy of the EMI emission. Furthermore, the constant variation in switching frequency complicates dynamic control algorithms, introducing a high degree of control complexity to the drive system.

Another option is to regulate the switching transient itself, which directly addresses the root physical cause of EMI generation. Passive methods, such as enlarging gate resistors or using heavy snubber circuits, slow the switching speed, but they also degrade system efficiency due to significant active-region power dissipation [20,21]. Conversely, active gate drive (AGD) technologies use closed-loop feedback to profile the dv/dt and di/dt trajectories during transitions [22,23,24,25]. Foundational research by F. Costa et al. [26] established that shaping pulse edges into S-shaped profiles (e.g., Gaussian [27] or cosine [28]) effectively concentrates spectral energy, suppressing high-frequency EMI more effectively than standard single-slope waveforms [4]. While subsequent studies [29,30] have attempted digital S-shape tracking, continuous active profiling demands extreme sub-nanosecond temporal resolution. In practical industrial environments, signal propagation delays, device nonlinearities, and thermal drift severely disrupt this delicate control, making typical AGD solutions too unreliable for motor drive deployment.

This paper addresses the multi-objective engineering conflict by proposing a discrete, source-level EMI suppression methodology that achieves active waveform shaping through the sequential gate driving of a series-connected IGBT topology. Deliberately introducing staggered delays between series devices reconstructs the typically abrupt output voltage edges into a deterministic, multi-level staircase profile. Importantly, rather than treating EMI as only an electrical compliance metric, this study uses an information-theoretic framework with entropy-based metrics, which have been shown to be highly effective for evaluating time series complexity in modern engineering disciplines [31], to assess electromagnetic emissions.

The primary contributions of this study are characterized by their ability to harmonize thermodynamic efficiency with informational order:

1. Algorithmic Simplicity and Low System Complexity: Unlike highly sensitive, continuous AGD systems, the proposed method uses the structural flexibility of series-connected IGBTs to shape edges. The method relies solely on robust, discrete, sequential delay logic implemented via a standard CPLD, ensuring extremely low control complexity and high operational reliability.
2. Spectral Ordering and Targeted Entropy Reduction: Discretizing the switching transient into defined staircase steps introduces deliberate structural periodicity into the output waveform. This mathematical reconstruction transforms stochastic, broadband, high-frequency EMI into an ordered, predictable harmonic spectrum, thereby reducing the system’s Normalized Spectral Entropy.
3. Decoupling Informational and Thermodynamic Entropy: Traditional passive edge-slowing mechanisms suppress spectral noise at the severe cost of generating thermal entropy, i.e., increased switching losses. The proposed sequential commutation strategy mitigates high-frequency informational chaos (EMI) without prolonging the total active-region switching transition. This achieves a rare and highly favorable balance between spectral entropy and energy efficiency.
4. Universal Compatibility: Informational optimization of the pulse edges occurs exclusively at the micro-transient level, entirely decoupled from the macroscopic PWM strategy. This ensures the methodology acts synergistically with conventional physical EMI filters and can be adapted to motor drives of various power classifications and duty cycles.

Following the Introduction, Section 2 analyzes the impact of voltage shape on the EMI spectrum and establishes a harmonic suppression model for staircased voltage shaping. Section 3 provides a detailed account of the implementation of a staircased voltage waveform using simple gate drive control. Section 4 experimentally validates source-level EMI suppression and reduced switching losses. Section 5 concludes the paper.

## 2. Source-Level EMI Suppression Mechanism

### 2.1. Effect of Voltage Shape on the EMI Spectrum

The voltage and current waveforms within the switching unit are the primary source of EMI. During switching transients, high d*v*/d*t* values generate significant displacement currents through parasitic capacitance coupling. These currents are the main cause of both conducted and radiated EMI in motor drive systems [4,26]. Therefore, the pulse edge and spectral components of the switching voltage directly determine the amplitude of the high-speed noise currents induced in the system [32,33]. Time-domain and frequency-domain analyses of the switching voltage are fundamental to understanding and suppressing electromagnetic interference [34].

**Figure 2:**
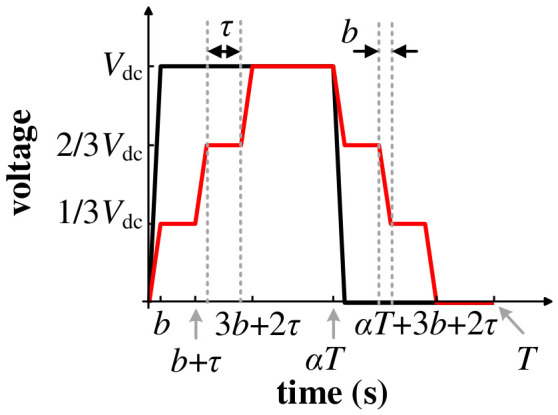
Comparison of conventional trapezoidal (red) and proposed staircased output voltage pulses (black).

As shown by the black curve in Figure 2, the inverter output voltage pulse approximates a trapezoidal wave with steep edges. This waveform comprises four phases: a rising edge, a high-level plateau, a falling edge, and a low-level plateau. Here, *A* represents the amplitude, *T* the period, and *α* the duty cycle. The rise/fall time is equal to *b*. This waveform can be represented as a piecewise function and expressed in terms of a frequency spectrum using a Fourier series expansion [35].

The Fourier coefficients *a*_0_, *a_n_* and *b_n_* are given by (1) and (3), which can be substituted into (4) to yield the Fourier series expansion.

(1) $$a_{0}=V_{\mathrm{dc}}\alpha$$

(2) $$a_{n}=\frac{-V_{\mathrm{dc}}T\left[\cos\left(\frac{2\pi n\left(b+\alpha T\right)}{T}\right)-\cos\left(\frac{2\pi bn}{T}\right)-\cos\left(2\pi \alpha n\right)+1\right]}{2\pi^{2}bn^{2}}$$

(3) $$b_{n}=\frac{V_{\mathrm{dc}}T\left[\sin\left(2\pi bn/T\right)-\sin\left(2\pi n\left(b+\alpha T\right)/T\right)+\sin\left(2\pi \alpha n\right)\right]}{2\pi^{2}bn^{2}}$$

(4) $$f\left(t\right)=a_{0}+\sum_{n=1}^{\infty }\left(a_{n}\cos2\pi nft+b_{n}\sin2\pi nft\right)$$

The red curve in Figure 2 shows the proposed staircased output voltage pulse. Shaping the edges into a staircased profile alters the spectral distribution, thereby suppressing EMI. In the circuit implementation, the number of voltage steps *N* equals the number of series-connected IGBTs per branch, with *N* set to 3. Each step lasts for a time period of *τ*, with the other parameters *V*_m_, *T*, *α*, and *b* matching the trapezoidal waveform. The Fourier coefficients *A*_0_, *A_n_*, and *B_n_*, defined in (5)–(7), characterize the spectral composition.

(5) $$A_{0}=-V_{\mathrm{dc}}\left(5b+2\tau -\alpha T\right)/T$$

(6) $$A_{n}=\frac{V_{\mathrm{dc}}T}{6b\pi^{2}n^{2}}\left\{\begin{array}{l} \cos\left(2\pi n\left(3b+2\tau \right)/T\right)+\cos\left(2\pi n\left(2b+\alpha T+2\tau \right)/T\right)\\ -\cos\left(2\pi n\left(3b++\alpha T+2\tau \right)/T\right)-\cos\left(2\pi n\left(b+\tau \right)/T\right)\\ +\cos\left(2\pi n\left(2b+\tau \right)/T\right)-\cos\left(2\pi n\left(2b+\alpha T+\tau \right)/T\right)\\ +\cos\left(2\pi n\left(b+\alpha T+\tau \right)/T\right)-\cos\left(2\pi n\left(b+\alpha T\right)/T\right)\\ -2\cos{\left(2\pi n\left(b+\tau \right)/T\right)}^{2}+\left(6bn\sin\left(2\pi \alpha n\right)\right)/T\\ -\left(6bn\sin\left(2\pi n\left(3b++\alpha T+2\tau \right)/T\right)\right)/T+\cos\left(2\pi \alpha n\right)\\ -\left(12b\pi n\sin\left(2\pi n\left(b+\alpha T\right)/T\right)\right)/T+\cos\left(2\pi nb/T\right) \end{array}\right\}$$

(7) $$B_{n}=\frac{V_{\mathrm{dc}}T}{6b\pi^{2}n^{2}}\left\{\begin{array}{l} \sin\left(2\pi n\left(b+\alpha T\right)/T\right)-\sin\left(2\pi n\left(3b+2\tau \right)/T\right)\\ -\sin\left(2\pi n\left(2b+2\tau +\alpha T\right)/T\right)+\sin\left(4\pi n\left(b+\tau \right)/T\right)\\ +\sin\left(2\pi n\left(3b+2\tau +\alpha T\right)/T\right)+\sin\left(2\pi n\left(b+\tau \right)/T\right)\\ -\sin\left(2\pi n\left(3b+\tau +\alpha T\right)/T\right)-\sin\left(2\pi bn/T\right)\\ -\sin\left(2\pi n\left(2b+\tau \right)/T\right)+\sin\left(2\pi n\left(2b+\tau +\alpha T\right)/T\right)\\ \left(-6\pi bn\cos\left(2\pi n\left(3b+2\tau -\alpha T\right)/T\right)\right)/T \end{array}\right\}$$

The complete series expansion is presented in (8).

(8) $$g\left(t\right)=A_{0}+\sum_{n=1}^{\infty }\left(A_{n}\cos2\pi nft+B_{n}\sin2\pi nft\right)$$

**Figure 3:**
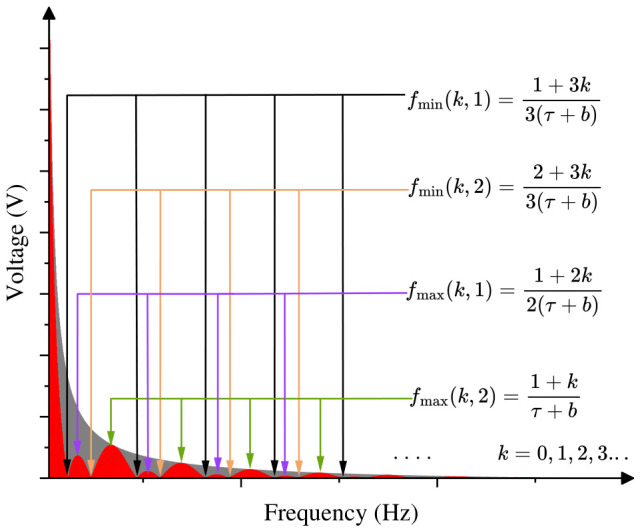
Spectral comparison between the conventional trapezoidal (gray) and the proposed staircased voltage (red).

Figure 3 compares the frequency spectra of the two voltage waveforms defined by (4) and (8). The gray curve represents the spectrum of the conventional trapezoidal waveform, and the red curve represents the staircased waveform. The trapezoidal waveform can be seen to exhibit a smooth spectral envelope, whereas the staircased waveform shows an oscillatory envelope with significantly better overall suppression. The staircase waveform’s spectrum displays periodic attenuation, with each period containing two peaks and two troughs. The attenuation period is 1/(*τ* + *b*) Hz. The peak and trough frequencies, denoted as *f*_max_ and *f*_min_, are given by the following expressions.

First trough frequency group:

(9) $$f_{\min}\left(k,1\right)=\left(1+3\left(k-1\right)\right)/3\left(\tau +b\right)\;\;k=1,2,3\ldots$$

Second trough frequency group:

(10) $$f_{\min}\left(k,2\right)=\left(2+3\left(k-1\right)\right)/3\left(\tau +b\right)\;\;k=1,2,3\ldots$$

First peak frequency group:

(11) $$f_{\max}\left(k,1\right)=\left(1+2\left(k-1\right)\right)/2\left(\tau +b\right)\;\;k=1,2,3\ldots$$

Second peak frequency group:

(12) $$f_{\max}\left(k,1\right)=\left(1+\left(k-1\right)\right)/\left(\tau +b\right)\;\;k=1,2,3\ldots$$

The parameters *τ* and *b* are crucial in determining the EMI attenuation performance. It is important to note that the initial attenuation bandwidth is less than 1/(*τ* + *b*) Hz, beginning at the knee frequency of the passband asymptote, which occurs at 1/(*π* (*αT* − *b*)) Hz. This indicates that the duty cycle *α* primarily influences the low-frequency spectral envelope and has a negligible impact on the high-frequency region [36]. Consequently, the proposed scheme remains applicable to applications requiring duty cycle adjustment. This section analysis emphasizes the vital role of staircase pulse edges in reducing high-frequency EMI. Subsequent sections discuss practical limitations associated with these parameters in real-world circuits, as well as their combined impact on the spectral envelope.

### 2.2. Periodic Source-Level EMI Suppression of Inverters

Previous analysis has indicated that reshaping voltage pulse edges into a staircased profile can effectively alter the spectral distribution and significantly reduce EMI. This section aims to derive the harmonic attenuation characteristics of the inverter output voltage based on staircase waveform reshaping. The focus is on how the key parameters, order *N* and step width *τ*, regulate the suppression effect and the distribution of specific harmonic components.

The output voltage of an inverter using sinusoidal pulse width modulation (SPWM) consists of a periodic sequence of voltage pulses with fixed amplitudes and modulated widths according to a sinusoidal pattern. Consequently, the duty cycle, *α*, is not constant but varies as a function of time, as expressed below.

(13) $$\alpha (t)=1+m\sin\left(\omega_{m}t\right)/2$$

where *m* represents the modulation index and *ω*_m_ equals 2*πf*_m_, signifying the angular frequency. *f*_m_ and *f*_c_, equal to 1/*T*_c_, represent the modulation frequencies and carrier frequencies, respectively. During the modulation process, the duty cycle is sampled and held constant over each carrier period *T*_c_. Assuming sampling occurs at time instants *t_n_* = *nT*_c_, the sampled duty cycle is denoted as *α_n_*.

(14) $$\alpha_{n}=\alpha \left(nT_{c}\right)=1+m\sin\left(\omega_{m}nT_{c}\right)/2$$

Represent the Fourier series for the trapezoidal and staircased waveforms with a specified duty cycle *α_n_* as *f* (*t*, *α_n_*) and *g* (*t*, *α_n_*), respectively. The entire SPWM output voltage waveform can then be expressed as a linear combination of voltage pulses, all of which have the same fundamental period but different duty cycles. The Fourier series expansions for the trapezoidal and staircased SPWM output voltages can therefore be obtained as shown in (15) and (16).

(15) $$h\left(t\right)=\sum_{n=-\infty }^{+\infty }f_{n}\left(t-nT_{c},\alpha_{n}\right)$$

(16) $$s\left(t\right)=\sum_{n=-\infty }^{+\infty }g_{n}\left(t-nT_{c},\alpha_{n}\right)$$

Due to the time-invariant property of the output voltage spectrum, the specific distribution of the duty cycle sequence *α_n_* within one fundamental period does not affect the overall envelope characteristics of the spectrum. As previously mentioned, the duty cycle primarily influences the knee frequency within the passband, while having a negligible effect on the attenuation envelope shape for high-frequency harmonics. The high-frequency harmonic attenuation envelope is primarily determined by three parameters: *τ*, *b* and *N*. These parameters control both the number of peaks and troughs and their bandwidth distribution characteristics.

(17) $$f_{\max}\left(k,h\right)=\frac{h+N\left(k-1\right)}{N\left(\tau +b\right)},h\in \left[1,N-1\right],k=1,2,3\cdots$$

(18) $$f_{\min}\left(k,h\right)=\frac{h+\left(N-1\right)\left(k-1\right)}{\left(N-1\right)\left(\tau +b\right)},h\in \left[1,N-1\right],k=1,2,3\cdots$$

**Figure 4:**
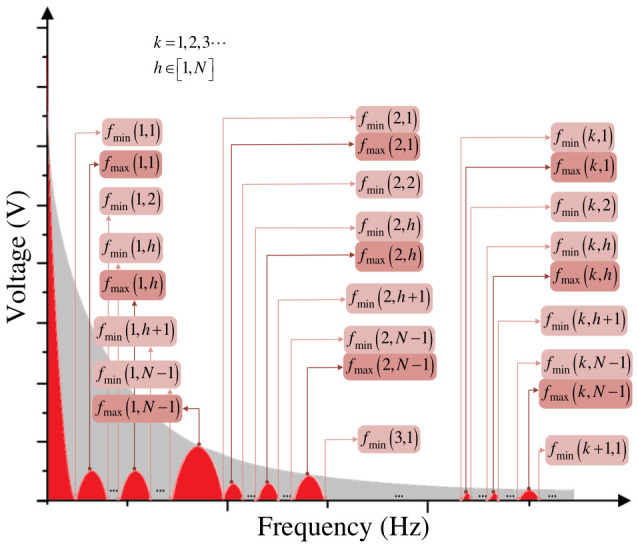
Frequency spectra of the conventional trapezoidal output voltage (gray) and proposed stepped (red) output voltage for inverters.

Figure 4 shows a comparison of the spectra of the trapezoidal and staircased voltages of the inverter. As (17) and (18) show, the spectral envelope of the staircased voltage contains *N* − 1 peaks and *N* − 1 troughs within each suppression band. The frequencies at the peaks and troughs are denoted as *f*_max_ (*k*, *h*) and *f*_min_ (*k*, *h*), respectively. Here, *k* represents the *k*-th suppression band, and h indicates the relative position of the peak/trough within that band. At the trough frequencies *f*_min_ (*k*, *h*), the harmonic components are suppressed to nearly zero. Although the attenuation at the peak frequencies *f*_max_ (*k*, *h*) is less pronounced than at the troughs, the spectral amplitudes at these peaks are consistently lower than those of the conventional trapezoidal voltage.

The described harmonic suppression characteristics provide a clear basis for designing EMI filters for inverters. Using an appropriate number of series devices and controlling the step duration can significantly reduce EMI within specific frequency bands.

### 2.3. Spectral Entropy Analysis of the Shaping Mechanism

To justify the proposed scheme within an information-theoretic framework further, the concept of Normalized Spectral Entropy (*H*_se_) is used to quantify the distribution of the EMI source. The Normalized Spectral Entropy provides a dimensionless index bounded between 0 and 1, where a value closer to 1 indicates a disordered, wideband noise distribution (resembling white noise), and a value closer to 0 indicates a more ordered, deterministic spectral structure. It is defined as

(19) $$H_{\mathrm{se}}=-\frac{1}{\ln K}\sum_{n=1}^{K}P(f_{n})\ln[P(f_{n})]$$

where *K* represents the total number of frequency bins within the analyzed bandwidth, and *P*(*f_n_*) is the normalized Power Spectral Density (PSD) at frequency component *f_n_*. The calculation of *H*_se_ treats the normalized power distribution of the inverter output as a probability density function. This approach aligns with recent methodologies used to characterize the information complexity of discrete frequency distributions, where Shannon entropy is utilized to distinguish between stochastic noise and structured signals [37].

The transition from a conventional trapezoidal waveform to the proposed staircased waveform represents a fundamental shift in the system’s entropy state:

Spectral ordering: While the conventional trapezoidal waveform exhibits a smooth spectral envelope that spreads harmonic energy broadly, the staircased waveform introduces a deliberate oscillatory envelope. As derived in (17) and (18), this structure creates *N* − 1 deterministic peaks and troughs within each suppression band. By concentrating energy into these predictable periodic patterns, the sequential switching control effectively imposes order upon the spectrum.

Information redundancy via periodic suppression: The existence of specific trough frequencies where harmonic components are suppressed to negligible levels signifies a reduction in the uncertainty of the EMI environment. This periodic nature increases the predictability of the system, which is mathematically equivalent to a reduction in Shannon entropy.

This analysis demonstrates that the shaping mechanism fundamentally alters the information content and energy distribution of the inverter’s electromagnetic emissions.

## 3. Implementation of Staircased Output Voltage

**Figure 5:**
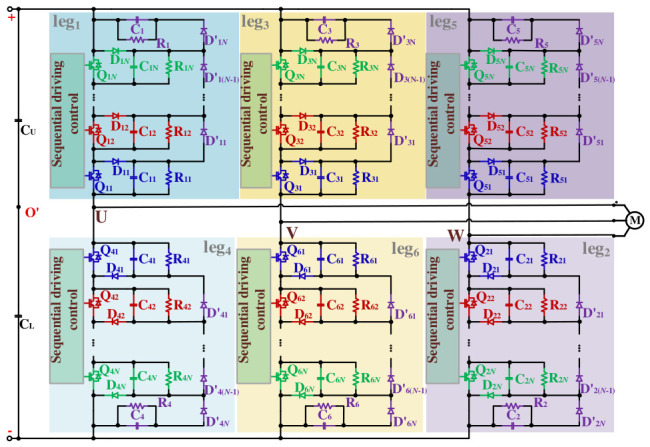
Schematic structure of the three−phase inverter of this paper.

Figure 5 shows the circuit topology of the proposed three-phase inverter. Each of the upper and lower switching legs, referred to as leg*_i_* and leg*_j_*, comprises *N* series-connected IGBTs, Q*_ix_* and Q*_jx_*, where *x* ranges from 1 to *N* and indicates the position of the IGBT relative to the phase midpoint.

Each IGBT Q*_ix_*/Q*_jx_* is connected in parallel with an RCD snubber circuit consisting of a static equalizing resistor R*_ix_*, a snubber capacitor C*_ix_*, and a decoupling diode D*_ix_*. The resistance of R*_ix_* typically ranges from several tens to several hundreds of kilohms, which is considerably lower than the IGBT’s leakage resistance in the off state. Consequently, the current through the equalizing resistor is significantly greater than the IGBT’s leakage current. This ensures that, in the off state, the voltage distribution across the IGBTs is dictated by R*_ix_* rather than by the varying equivalent leakage resistances of the IGBTs, thus achieving static voltage balancing. During the turn-off process, any surplus energy resulting from voltage imbalance due to discrepancies in dynamic parameters is directed through D*_ix_* to C*_ix_*. This suppresses overvoltage, providing dynamic voltage balancing and device protection. The next section explains how these components work together to shape a staircase waveform from the output voltage pulses.

### 3.1. Sequential Gate Drive Control Circuit

In industrial motor drive applications, the complexity of inverter control can result in problems such as delayed dynamic responses, reduced reliability, and increased costs. This paper proposes an output-voltage-shaping scheme based on a series-connected IGBT structure. This approach only requires sequential switching control via the CPLD in the traditional drive circuit, offering simple and flexible operation. Specific time delays are introduced to each phase’s SPWM signal, enabling the IGBTs within the same branch to be triggered sequentially and forming a staircase-edge voltage pulse waveform. This design effectively improves the spectral characteristics of the output voltage without increasing control complexity.

**Figure 6:**
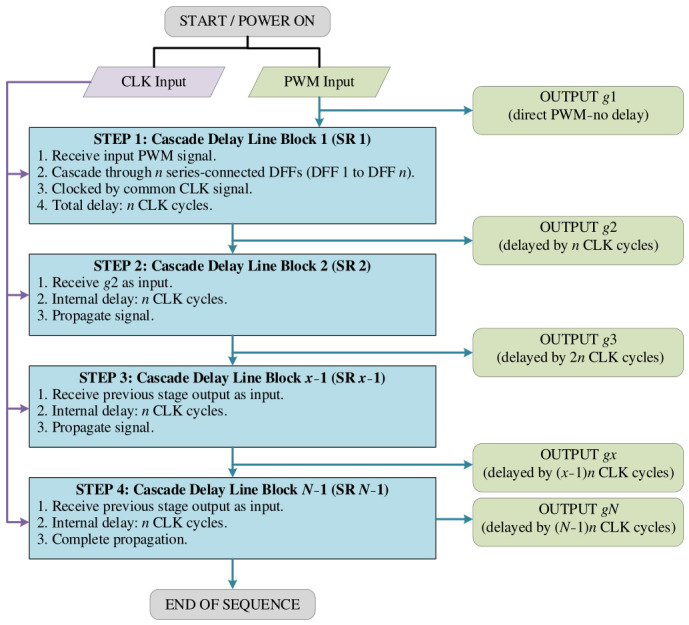
Functional block diagram of the sequential gate drive delay mechanism.

The functional block diagram of the sequential gate drive delay mechanism is illustrated in Figure 6. The primary PWM input signal is directly mapped to the first output, *g*1, with no delay. Simultaneously, the PWM signal is fed into a cascade delay line comprising *N* − 1 shift register blocks (SR 1 to SR *N* − 1). The first block (SR 1) contains *n* series-connected D flip-flops (DFFs). Triggered by a common CLK signal, this block introduces a base delay of *n* clock cycles to generate the second output, *g*_2_. This delayed signal then propagates through the subsequent stages, with each block acting as the input for the next and introducing an additional identical delay. Consequently, an intermediate output signal *g_x_* undergoes a cumulative time delay of (*x* − 1) *n* clock cycles, and the final stage outputs *g_N_* with a total delay of (*N* − 1) *n* clock cycles. Assuming the time delay introduced by a single block is Δ*t* = *n* × *T*_clk_ (where Δ*t* = *τ* + *b*), these cascaded shift registers successfully implement the staggered sequential switching control of the series-connected IGBTs. Precise timing control adjusts the duration of each voltage step *τ* and shapes the edge characteristics of the staircased voltage waveform.

### 3.2. Staircased Voltage Shaping Process by Sequential Gate Drive

In a three-phase inverter system feeding an asynchronous motor, the load exhibits pronounced inductive characteristics. Consequently, during the switching transitions of the IGBTs, the load current *I*_L_ behaves essentially as a constant current source to maintain continuity. In a conventional series-connected IGBT topology without auxiliary circuits, turning off a single device interrupts this inductive current, inducing overvoltage and transitioning the entire leg into the off-state.

To achieve controllable multi-level voltage shaping, the proposed scheme utilizes the interaction between the continuous inductive load current and the parallel RCD snubber network R*_ix_*, C*_ix_*, and D*_ix_*. When the sequential gate drive turns off the first IGBT (e.g., Q*_i_*_1_), the inductive nature of the load maintains current continuity. The current *I*_L_ is therefore commutated to the parallel snubber capacitor C*_i_*_1_. As C*_i_*_1_ charges, the voltage across Q*_i_*_1_ rises to its equalized share *V*_dc_/*N*, while the remaining *N* − 1 IGBTs in the branch remain in the conduction state. This causes the output phase voltage *V*_UO_ to decrease by *V*_dc_/*N*, generating a single voltage step rather than a complete voltage drop.

**Figure 7:**
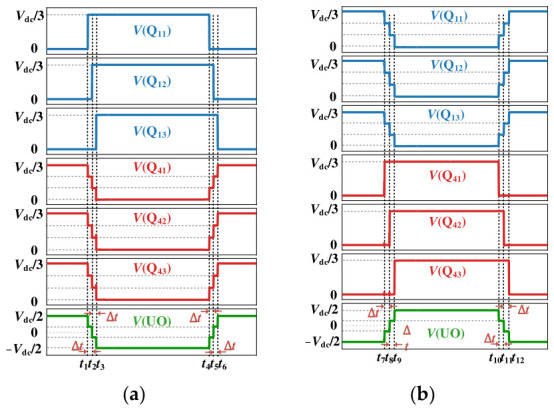
Voltage waveforms when (**a**) *I*_L_ > 0 and (**b**) *I*_L_ < 0.

By repeating this staggered switching process with a defined time delay Δ*t*, the load current is sequentially commutated to the subsequent snubber capacitors or freewheeling diodes, forming a staircase voltage waveform. The polarity of *I*_L_, combined with the sequential gate signals, determines the specific current commutation paths. The detailed switching mechanism is analyzed below for different load current directions, focusing on phase U. For example, consider the upper and lower legs, each of which is composed of three IGBTs connected in series. Figure 7 illustrates the voltage waveforms for *I*_L_ > 0 and *I*_L_ < 0 in the upper and lower legs. When *I*_L_ > 0, current flows from the inverter to the motor, and when *I*_L_ < 0, current flows from the motor to the inverter.

**Figure 8:**
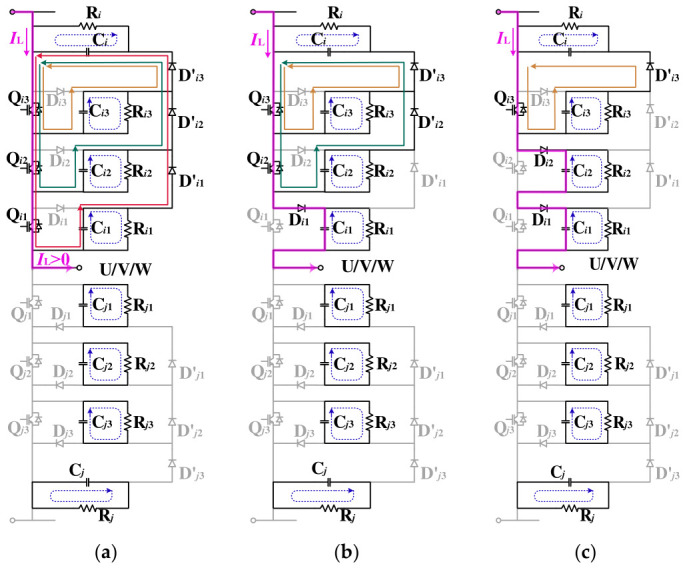

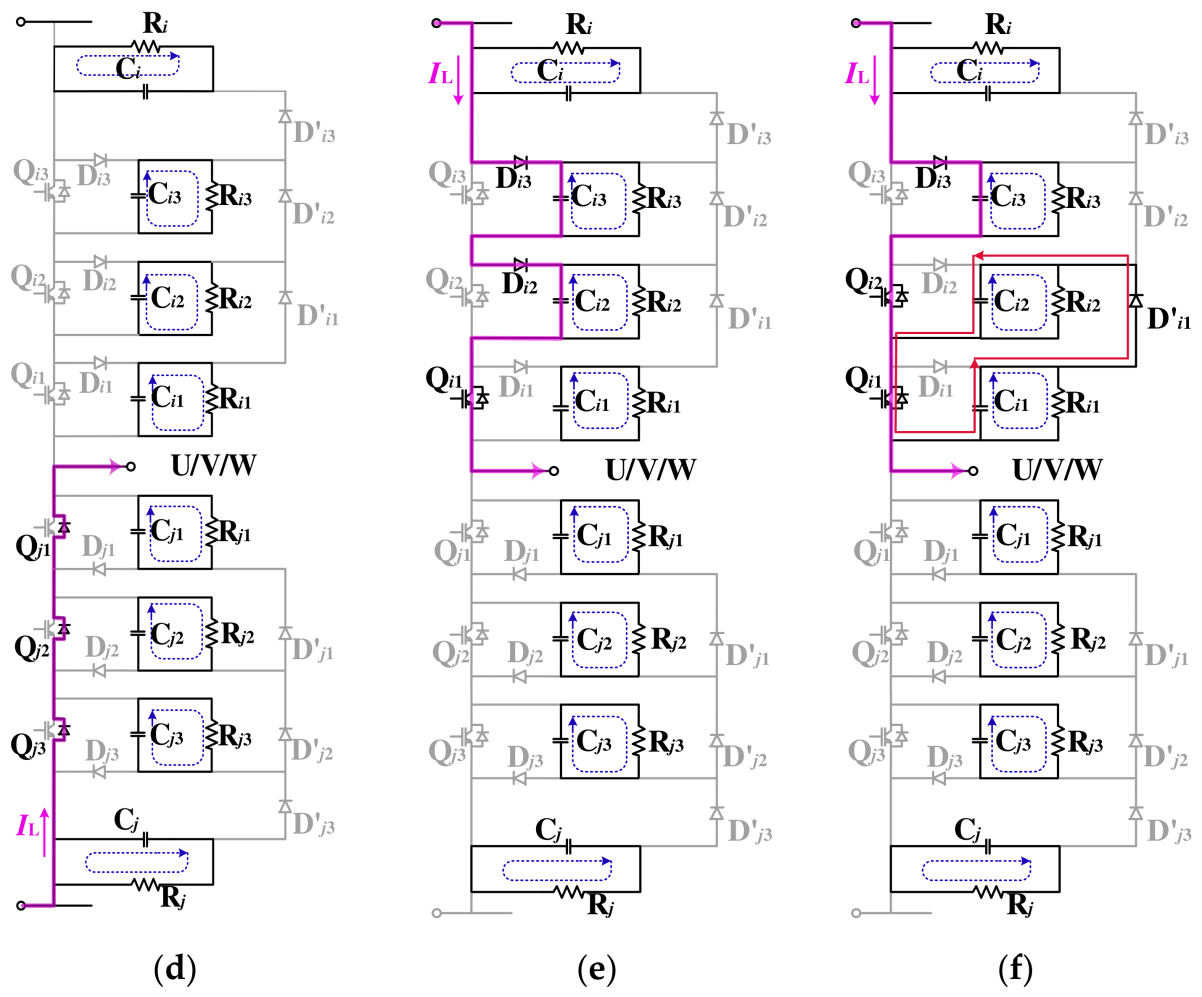
Sequential switch process when *I*_L_ > 0: (**a**) Q*_i_*_1_, Q*_i_*_2_ and Q*_i_*_3_ turn on and phase U is in state1. (**b**) Q*_i_*_1_ turns off. (**c**) Q*_i_*_1_ and Q*_i_*_2_ turn off. (**d**) Q*_i_*_1_, Q*_i_*_2_ and Q*_i_*_3_ turn off and phase U is in state0. (**e**) Q*_i_*_1_ turns on. (**f**) Q*_i_*_1_ and Q*_i_*_2_ turn on.

**Figure 9:**
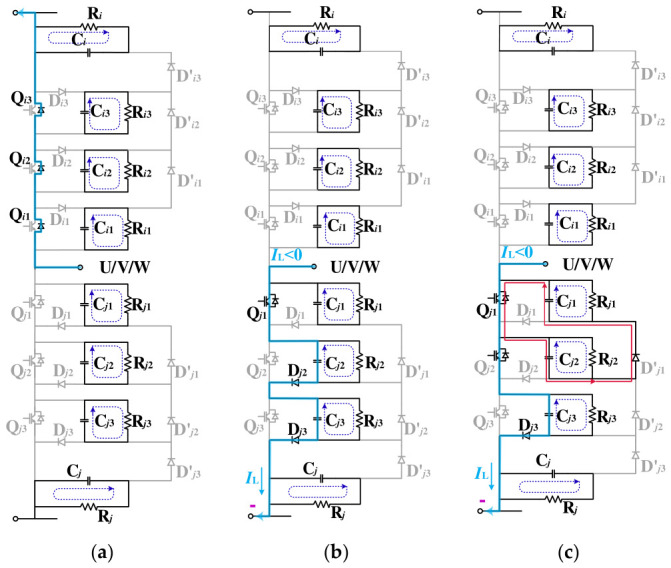

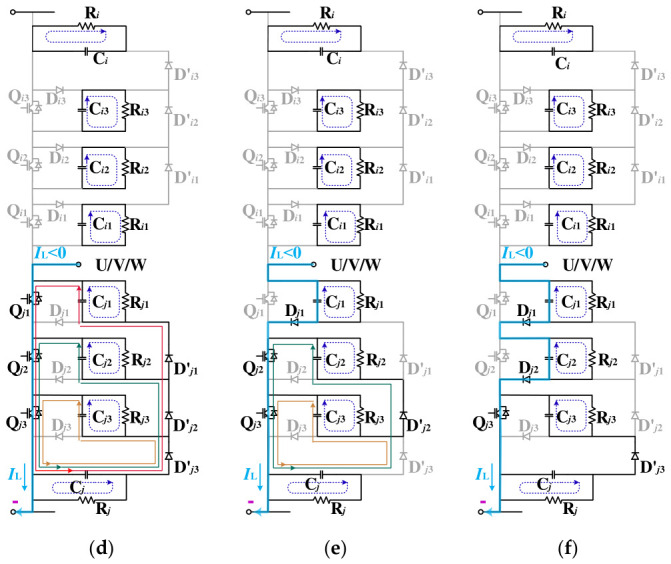
Sequential switch process when *I*_L_ < 0: (**a**) Q*_j_*_1_, Q*_j_*_2_ and Q*_j_*_3_ turn off and phase U is in state1. (**b**) Q*_j_*_1_ turns on. (**c**) Q*_j_*_1_ and Q*_j_*_2_ turn on. (**d**) Q*_j_*_1_, Q*_j_*_2_ and Q*_j_*_3_ turn on and phase U is in state0. (**e**) Q*_j_*_1_ turns off. (**f**) Q*_j_*_1_ and Q*_j_*_2_ turn off.

In Figure 7, the phase voltage *V*_UO_ is defined as the potential differences between the midpoints of phase U and the midpoint O of the DC-link capacitor. The switching states are defined according to standard convention. Figure 8 and Figure 9 show the current paths and the switching states of leg*_i_*/leg*_j_*, which can explain how sequential drives shape the voltage edge. The mechanism that forms the staircase voltage waveform is described below. State1 indicates activation of the upper leg and deactivation of the lower leg. State0 indicates activation of the lower leg and deactivation of the upper leg.

Case 1: When I_L_ > 0, Leg_1_ turns off sequentially from state1 to state0.

*t*_1_–*t*_3_: As shown in Figure 8a–d, the IGBTs in leg_1_ are turned off sequentially. Starting at time *t*_1_, the low-level drive signals *g_i_*_1_, *g_i_*_2_, and *g_i_*_3_ are sequentially delayed by Δ*t* in order to control the sequential turn-off of Q*_i_*_1_–Q*_i_*_3_. Assuming ideal voltage sharing, the voltage across each snubber capacitor C*_ix_* is *V*_dc_/3. Once Q*_i_*_1_ has been turned off, the load current switches to its parallel RCD snubber circuit. At this time, C*_i_*_1_ is being charged. The potential at node U decreases by *V*_dc_/3, meaning V_UO_ decreases by *V*_dc_/3. The IGBTs Q*_j_*_1_–Q_j3_ share this voltage change, with the *V*_ce,*jx*_ voltage across each one decreasing by *V*_dc_/9. The turn-off processes of Q*_i_*_2_ and Q*_i_*_3_ are similar to Q*_i_*_1_.

*t*_3_–*t*_4_ (State0): Once leg*_i_* has been fully switched off, the current path switches to the freewheeling diodes of Q*_j_*_1_–Q*_j_*_3_, as shown in Figure 8d.

Case 2: When I_L_ > 0, Leg_1_ turns on sequentially from state0 to state1.

*t*_4_–*t*_6_: The sequence of activation of the IGBTs in leg_1_ is illustrated in Figure 8d–f. From time t_4_ onwards, the high-level drive signals *g_i_*_1_, *g_i_*_2_, and *g_i_*_3_ are delayed sequentially by Δ*t* to control the turn-on of Q*_i_*_1_–Q*_i_*_3_ in sequence. Once Q*_i_*_1_ is fully turned on, D*_i_*_1_ decouples the snubber circuit. This increases the potential at node U by *V*_dc_/3, meaning V_UO_ also increases by *V*_dc_/3. This voltage increase is shared by the turned-off Q*_j_*_1_–Q*_j_*_3_, with *V_ce_*_,*jx*_ rising by *V*_dc_/9 across each one. The turn-on processes of Q*_i_*_2_ and Q*_i_*_3_ are analogous.

After *t*_6_ (state1): Once the upper arm becomes fully conductive, the current path switches back to it, as shown in Figure 8a. In state1, resistors R*_i_*_1_–R*_i_*_3_ are short-circuited and the diodes D*_i_*_1_–D*_i_*_3_ are reverse-biased, decoupling C*_i_*_1_–C*_i_*_3_ from the IGBTs.

Case 3: When I_L_ < 0, Leg_4_ turns on sequentially from state1 to state0.

*t*_7_–*t*_9_: As shown in Figure 9a–d, the turned-on sequence of the IGBTs in leg_4_ is from Q*_j_*_1_ to Q*_j_*_3_. When Q*_j_*_1_ is conducting but Q*_j_*_2_ and Q*_j_*_3_ are not, *I_L_* flows through R*_j_*_2_, D*_j_*_2_, C*_j_*_2_, R*_j_*_3_, D*_j_*_3_ and C*_j_*_3_. During this process, the capacitor stores energy. Once Q_j1_ and Q_j2_ have turned on, I_L_ flows through R*_j_*_3_, D*_j_*_3_ and C*_j_*_3_. The capacitor continues to store energy during this process until Q*_j_*_1_ to Q*_j_*_3_ are fully turned on and the U-phase transitions to state0, as shown in Figure 9d.

Case 4: When I_L_ < 0, Leg_4_ turns off sequentially from state0 to state1.

*t*_10_–*t*_12_: As Figure 9d–f show, the IGBTs in leg_4_ are turned off sequentially. When Q*_j_*_1_ is turned off first, the current path is interrupted and *I*_L_ flows through its snubber circuit. Next, Q*_j_*_2_ and Q*_j_*_3_ turn off sequentially. As the current direction cannot change abruptly, the current path shifts to the freewheeling diodes of the IGBTs in leg_1_.

The aforementioned switching control strategy successfully shaped the edges of the output voltage pulses into a staircase pattern during the inverter’s drive of the asynchronous motor.

### 3.3. Damping Resistors R_i_

In this paper, each switch leg uses a resistor to damp energy, generated by sequential switch. According to Figure 8 and Figure 9, during the sequential switching of the IGBTs, the current charges C*_ix_* and C*_jx_*. Taking leg_1_ as an example, once it is in the on state, components Q_11_–Q_13_, C_11_–C_13_, D′_11_–D′_13_, and capacitor C_1_ form three current paths, as shown in Figure 8a. Excess energy stored in C_11_-C_13_ can be transferred to C_1_. R*_i_* prevents C*_i_* from saturating and prepares for the next sequential switching by discharging C*_i_*.

## 4. Experimental Verification

### 4.1. Experimental Setup Prototype

**Figure 10:**
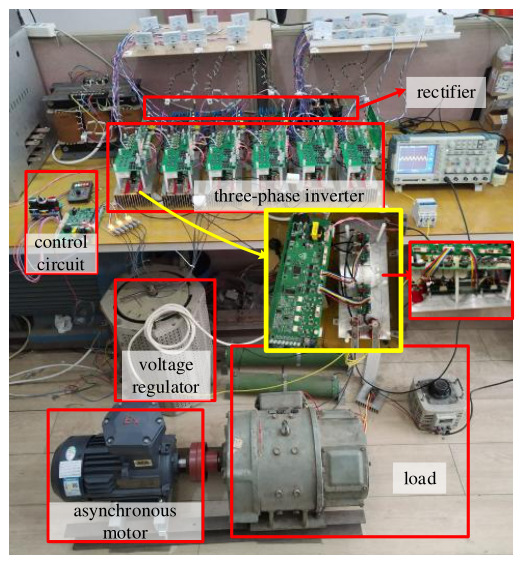
Prototype of the experimental setup.

A 1.14 kV three-phase inverter prototype has been constructed experimentally to validate the performance of sequential switching control. Figure 10 shows the experimental setup prototype. The three-phase inverter comprises four IGBTs connected in series in each switch leg. The load used in this experiment is a flameproof three-phase asynchronous motor, designed for use in mines. The experimental specifications are outlined in Table 1.

The choice of *N* = 4 series-connected IGBTs per switch leg in the experimental prototype is determined by the DC-link voltage and the voltage rating of the selected commercial devices (IKW20N60H3, rated at *V*_CES_ = 600 V). Under ideal steady-state voltage sharing, each device withstands 375 V during the off state, providing a voltage safety margin of approximately 37.5% to accommodate dynamic voltage imbalances during transients.

### 4.2. Experimental Verification of Voltage-Shaping Capability

**Figure 11:**
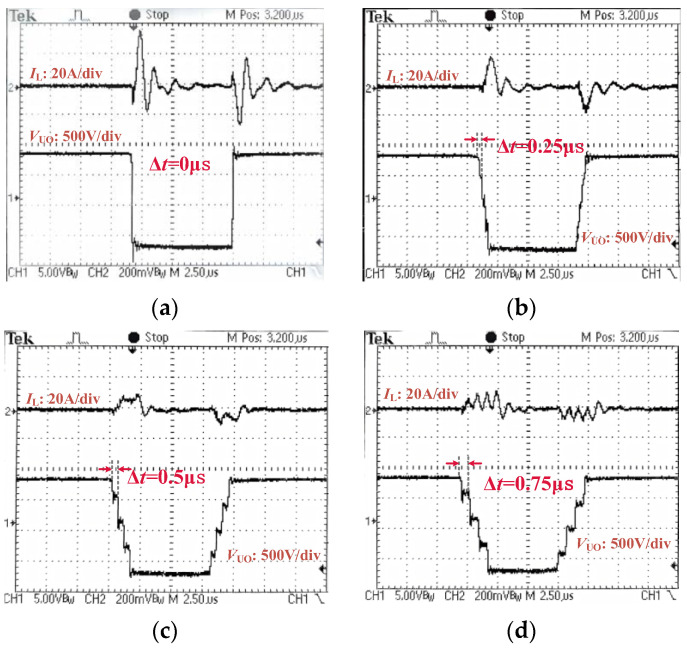
Phase voltage and current waveforms under synchronous switching and sequential switching: (**a**) Δ*t* = 0 μs (synchronous switching), (**b**) Δ*t* = 0.25 μs, (**c**) Δ*t* = 0.5 μs, (**d**) Δ*t* = 0.75 μs.

Figure 11 shows the voltage and current waveforms for phase U when the phase current *I*_U_ is positive and for different delay times Δ*t*. Figure 11a–d present the corresponding experimental results for delay times of 0 μs, 0.25 μs, 0.5 μs, and 0.75 μs, respectively.

As shown in Figure 11, when Δ*t* = 0 μs, the inverter is equivalent to a conventional two-level inverter. As outlined in the operational principles, the falling edge of V_UO_ signifies the turn-off process of leg_1_, while the rising edge corresponds to the turn-on process. Once the delay time reaches 0.5 μs, it is evident that a distinct staircase profile starts to appear in the output voltage waveform. The value of Δ*t* is crucial in influencing the voltage-shaping effect, as its efficacy depends on the relationship between Δ*t* and the time required for the collector-emitter voltage *V*_ce_ to complete its transition during IGBT switching. This time is indicated as parameter *b* in Figure 2. If Δ*t* is less than b, the switching processes of the series-connected IGBTs will overlap in the active region, resulting in severe voltage imbalance. This can cause the IGBTs to experience voltages significantly higher than their rated capacity, posing a high risk of overvoltage breakdown and placing high demands on the circuit protection design. Consequently, risks are only mitigated, and effective staircase voltage shaping is only achieved, when Δ*t* exceeds *b*.

**Figure 12:**
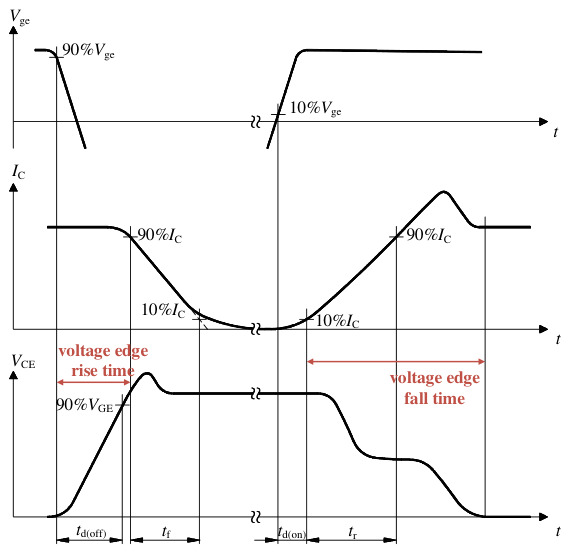
Definition of switching times of IGBT.

Figure 12 and Table 2 provide the definitions and specific values of the switching characteristic parameters of the IGBTs. The switching process of an IGBT essentially involves the charging and discharging of its internal parasitic capacitors. This process requires a finite duration and cannot occur instantaneously. The turn-off delay time is defined as the interval between the gate-emitter voltage *V*_ge_ decreasing to 90% of its initial value and the collector-emitter voltage *V*_ce_ rising to 90% of its final value. This period is primarily used for discharging the gate capacitor until the voltage falls below the threshold required to maintain the channel. The turn-on delay time refers to the duration from when the gate drive voltage increases to 10% of *V*_ge_ until *V*_ce_ begins to fall, which corresponds to the collector current *I*_c_ rising to 10% of its nominal value. This process primarily involves charging the gate-emitter capacitor C_ge_ until the threshold voltage is achieved.

Based on the provided parameters, the voltage edge rise time during the turn-off process for the IGBT used in this inverter is approximately 0.5 μs. The voltage fall time during the turn-on process is influenced by the Miller plateau and is difficult to determine precisely. Therefore, to produce a distinct staircase shape in the voltage waveform’s rising and falling edges of the voltage waveform, the delay time Δ*t* must exceed 0.5 μs. The actual switching time characteristics provide essential guidance for configuring the delay time.

### 4.3. Bounds on Delay Time Estimation in Practical Circuits

Once the minimum delay time necessary for generating the staircase waveform has been determined, the selection of Δ*t* in a practical inverter circuit must consider various constraints, such as the number of IGBTs connected in series and the SPWM control timing. This section examines the feasible range of Δ*t* based on the operating characteristics of a series-connected IGBT inverter under SPWM control.

With the SPWM control strategy, the voltage pulse width varies dynamically with the instantaneous value of the modulation wave. The pulse width is at its minimum when the carrier wave coincides with the modulation wave’s peak. This minimum pulse width, denoted as *t*_p_, can be calculated using (20).

(20) $$t_{p}\approx \left(1-m\right)/2f_{c}$$

Since the voltage fall time during IGBT turn-off is usually longer than the voltage rise time during turn-on, the delay time Δ*t* must satisfy the constraint given by (21). This ensures that all the IGBTs in the branch can complete the turn-off process within the time interval of *τ*, even under the minimum pulse condition. Here, *b* denotes the output voltage fall time. Substituting (20) into (21) yields the upper limit of the delay time, as shown in (22).

(21) $$b+\left(N-1\right)\Delta t\leq t_{p}$$

(22) $$\Delta t\leq \left(1-m-2bf_{c}\right)/\left(2f_{c}\left(N-1\right)\right)$$

In industrial inverter-fed motor applications, the carrier frequency usually falls within the range of a few to tens of kilohertz. Consequently, the limit defined by (22) is relatively broad. However, an excessively long delay time would considerably prolong the total switching process of the series-connected IGBTs, thereby increasing switching losses and reducing system efficiency. Therefore, to maintain a balance between system stability and conversion efficiency, it is advisable to avoid excessively long delays. Based on engineering practice, the total delay time of all IGBTs should be no more than 3% of the carrier period *T*_c_. Therefore, Δ*t* must also conform to the constraint defined by (23).

(23) $$b<\Delta t<\min(3\%T_{c}/N,\left(1-m-2bf_{c}\right)/\left(2f_{c}\left(N-1\right)\right))$$

In summary, the feasible range of Δ*t* is subject to multiple constraints. Subsequent experiments examine the relationship between Δ*t* and the frequency of harmonic attenuation.

### 4.4. Analysis of Source-Level EMI Suppression with Staircased-Shaped Voltage

**Figure 13:**
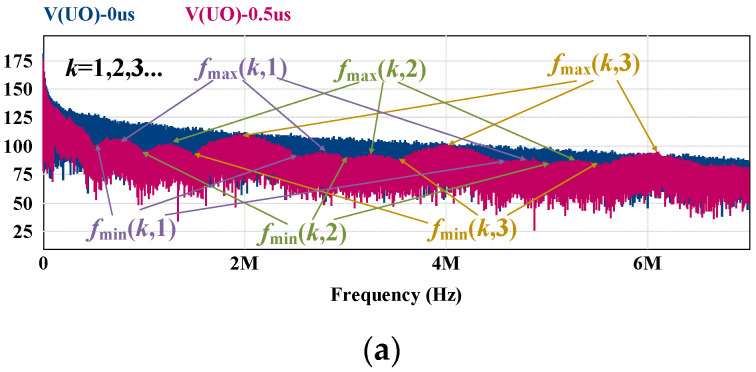

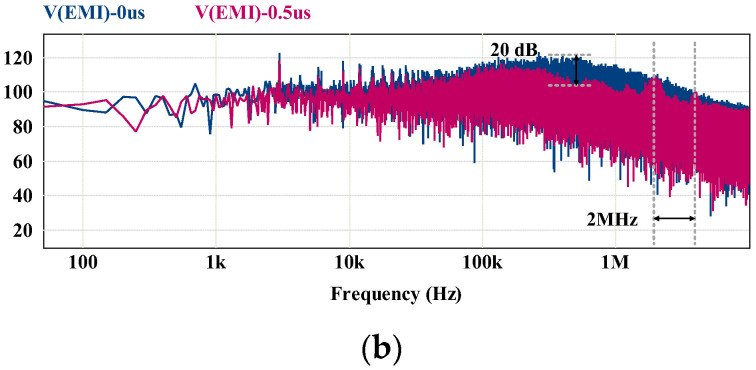
Simulated spectra under synchronous switching (Δ*t* = 0 μs, blue) and sequential switching (Δ*t* = 0.5 μs, red): (**a**) phase voltage spectrum; (**b**) EMI spectrum.

Figure 13a,b show the phase voltage spectra for synchronous and sequential switching modes on dBμV scales, as well as the EMI spectrum. The results indicate that the measured spectra align with the attenuation characteristics predicted by previous theoretical analysis. Compared to a traditional two-level inverter, the inverter based on staircased voltage shaping of series-connected IGBTs results in the periodic suppression of voltage harmonics. Based on the aforementioned limitation of delay time, a delay time of 0.5 μs was selected. When Δ*t* = 0.5 μs, the periodic suppression bandwidth is 2 MHz. Each suppression period consists of three peaks and three troughs.

Table 3 shows the harmonics information of the staircased voltage and the trapezoidal voltage. Under sequential switching, the harmonics can be reduced by 3.4–31.9 dB during every periodic suppression bandwidth. These results are consistent with the analysis in Section 2.

**Figure 14:**
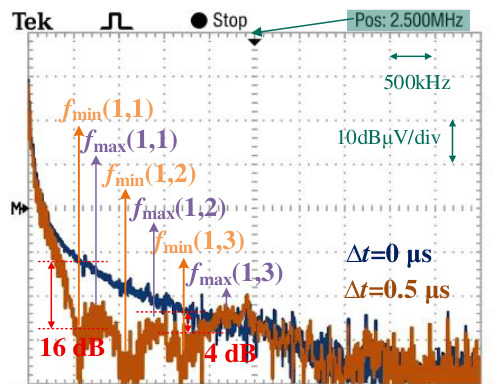
Experimental spectrum of the phase voltage *V*_UO_.

Figure 14 shows the spectra obtained by performing a fast Fourier transform on the phase voltage V_UO_ using an oscilloscope in synchronous and sequential switching control. Introducing a 0.5 μs delay reduces the harmonics within the first suppression period by 4–16 dB. The experimental results demonstrate that the staircase shaping creates a highly ordered spectral structure, leading to a significant reduction in spectral entropy. This manifests as a 4–16 dB μV attenuation, transforming a chaotic noise floor into a predictable harmonic pattern. Beyond 2.5 MHz, the spectral characteristics become less pronounced due to constraints such as the oscilloscope’s resolution and the probe’s bandwidth. Nevertheless, as the spectrum is essentially a Fourier transform of the output voltage waveform, the pattern of harmonic suppression remains consistent with theoretical predictions. It is important to note that certain frequency deviations were detected during the experiments, primarily due to non-ideal aspects of the hardware circuit, such as delays in the control signal and variations in component parameters. However, these deviations did not significantly impact the EMI suppression capability of the staircased voltage, and the attenuation performance remained consistent with the forecasts.

**Figure 15:**
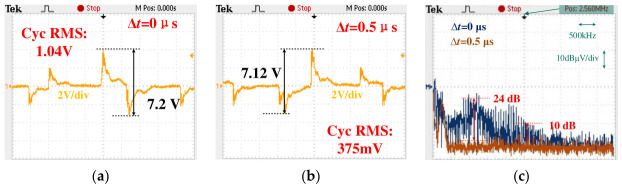
Measured EMI waveforms and their corresponding frequency spectra: The time-domain waveforms received by the antenna for (**a**) synchronous switching (Δ*t* = 0 μs) and (**b**) sequential switching (Δ*t* = 0.5 μs). (**c**) The frequency spectra of EMI for synchronous switching (Δ*t* = 0 μs) and sequential switching (Δ*t* = 0.5 μs).

Figure 15a–c show the measured EMI waveforms and frequency spectra. To validate the source-level EMI suppression scheme further, near-field radiation was captured near the motor drive system with a calibrated antenna and analyzed with a high-bandwidth oscilloscope. Figure 15a and Figure 15b show the EMI signal waveforms before and after the implementation of the output voltage shaping scheme, respectively. Key performance metrics, Cycle-RMS and Peak-to-Peak voltage (Pk-Pk), were recorded under two operating conditions. Cycle-RMS represents the effective value of AC within a single complete cycle and is a critical metric for measuring and comparing the average energy of periodic high-frequency noise. The experimental results demonstrate that this suppression technique reduced the cycle-RMS value from 1.04 V to 375 mV, achieving a 64% reduction. The reduction in Cycle-RMS values reflects a transition from a high-entropy noise state in synchronous switching to a more ordered, low-entropy state under sequential control. Meanwhile, the peak-to-peak voltage reduced from 7.2 V to 7.12 V. Figure 15c shows EMI suppression under sequential switching control. When combined with the inverter output phase currents shown in Figure 11, sequential switching control significantly suppresses high-frequency current spikes during switching transients. Mitigating these spikes reduces the high-frequency harmonic components that would otherwise couple into the system as common-mode and differential-mode noise.

To evaluate the effects of spectral order quantitatively, the Normalized Spectral Entropy *H*_se_ of the phase voltage *V*_UO_ and near-field EMI within a 2 MHz bandwidth was calculated. For the phase voltage, synchronous switching produced a highly disordered broadband spectrum with an *H*_se_ value of 0.9236. After adopting sequential switching (Δ*t* = 0.5 μs), *H*_se_ decreased to 0.8665. As *H*_se_ uses a logarithmic scale across thousands of frequency components, this reduction confirms a significant structural transformation. Harmonic energy has shifted from a chaotic, flat noise floor to a deterministic, periodic pattern with specific troughs and peaks. Similarly, the measured *H*_se_ value for near-field EMI decreased from 0.9102 to 0.8967.

The experimental results suggest that the reduction in external electromagnetic interference is due to improved internal waveform quality, which is achieved through source-level suppression measures. Although limitations of the instrument prevented the complete capture of detailed spectral characteristics in the high-frequency range, the available data clearly demonstrates the practical value of this method in suppressing electromagnetic interference. Subsequent analyses will incorporate switch loss evaluation to provide a comprehensive assessment of this topology’s performance in engineering applications.

Although the absolute decrease in EMI entropy is smaller than that of the phase voltage due to the inevitable injection of random spatial white noise and parasitic resonance frequencies during electromagnetic propagation, this sustained downward trend remains significant. It indicates that the structural order imposed by the source-level stepped voltage shaping is robust enough to effectively reduce the overall complexity and information chaos of the external electromagnetic environment.

### 4.5. Analysis of Switch Loss Under the Staircase Voltage Shaping

**Figure 16:**
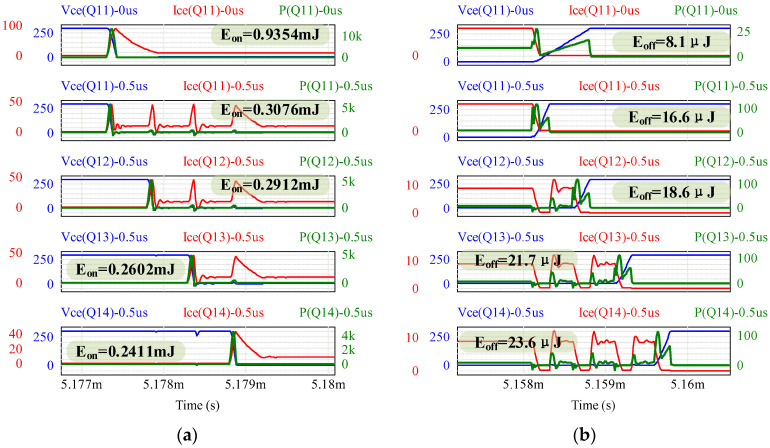
Voltage, current and instantaneous power waveforms of Q_11_–Q_14_ with and without sequential switching: (**a**) during turn-on transitions; (**b**) during turn-off transitions.

Figure 16a and Figure 16b respectively show the voltage, current and instantaneous power waveforms during the turn-on and turn-off transitions of IGBTs Q_11_ to Q_14_. The black curve represents the instantaneous power *p*. When Δ*t* is set to 0 μs, all the IGBTs operate synchronously; Q_11_ is selected for analysis; and Q_12_, Q_13_, and Q_14_ are the same as Q_11_. To accurately evaluate the change in switching loss following the implementation of the staircase output, the integration interval *t*_sw_ is defined as the time taken for leg_1_ to fully turn on and off. The turn-on loss *E*_on_ and turn-off loss *E*_off_, shown in Figure 16, are calculated using (24).

(24) $$E_{\mathrm{on}/\mathrm{off}-x}=\int_{t_{\mathrm{sw}}}P(t)dt=\int_{t_{\mathrm{sw}}}V_{\mathrm{ce}}(t)\cdot I_{\mathrm{ce}}(t)dt$$

During sequential turn-on, the phase voltage transitions from −*V*_dc_ to *V*_dc_ in four steps. This results in a significantly lower voltage change rate than with synchronous switching. Consequently, the current spike is substantially reduced. Each voltage step provides a current path for the IGBT that is subsequently turned on. However, since *V*_ce_ remains at the saturation level during this process, its value is very small. Consequently, the turn-on energy Eon of each IGBT is much lower than that in synchronous turn-on.

During the sequential turn-off process, each turn-off action instantly diverts part of the main current to charge the snubber capacitor, resulting in a notch appearing in the current waveform. After the turn-off, the current is redistributed among the remaining conducting devices. Consequently, the energy dissipated by each IGBT during the entire branch turn-off process is greater than during synchronous turn-off. These characteristics are consistent with the experimental results shown in Figure 16. The total equivalent switching loss *E_sw_* of the branch, calculated as the sum of the losses from Q_11_ to Q_14_ using (25), is 3.774 mJ for synchronous switching and just 1.1803 mJ for sequential switching. These results demonstrate that the staircase voltage method not only effectively suppresses EMI sources and significantly reduces switching losses.

(25) $$E_{\mathrm{sw}}=\sum_{x=1}^{4}\left(E_{\mathrm{on}-x}+E_{\mathrm{off}-x}\right)$$

### 4.6. Analysis of Inverter Losses Under Staircased Voltage Shaping

This section compares the power characteristics of the inverter under conventional two-level switching and the proposed staircase voltage shaping. The measured data is shown in Table 4. After shaping the output voltage into a staircase waveform, the active power increases by approximately 47.7 W compared to the conventional two-level inverter, primarily due to the additional loss introduced by the RCD snubber circuit. This accounts for only 1.59% of the rated power. Although the snubber circuit contributes to thermodynamic entropy generation through its 47.7 W loss, the overall system complexity is reduced by relaxing the requirements for secondary EMI filters.

Meanwhile, the reactive and apparent power increase by 15.4 VAR and 50.1 VA, respectively. This increase results from changes in the output voltage waveform under a constant load current. Specifically, the staircase edges, designed to suppress high-frequency EMI, redistribute harmonic energy from the suppressed high-frequency band to the low-frequency region. These low-frequency harmonic voltages interact with the load current, generating additional distorted reactive power and raising the total RMS voltage, which in turn increases the apparent power demand. Consequently, the power factor decreases slightly from 0.9973 to 0.9966. This marginal reduction aligns with the increased harmonic content, as the apparent power driven by harmonic voltages rises slightly faster than the active power, the latter being dominated by the fixed snubber loss.

In order to evaluate the system’s entropy state comprehensively, it is necessary to consider the reduction in information complexity (Normalized Spectral Entropy, *H*_se_) and the thermodynamic entropy generation rate (*S*_th_) introduced by the additional power consumption. Power loss from the RCD snubber circuit increases by 47.7 W, contributing to local thermodynamic entropy production.

(26) $$S_{\mathrm{th}}=\frac{P_{\mathrm{loss}}}{T_{\mathrm{amb}}}$$

where *T*_amb_ is the absolute ambient temperature. To maintain consistency with the normalized nature of *H*_se_, a comprehensive system entropy metric, *ψ*_sys_, is formulated using a baseline-ratio approach.

(27) $$\psi_{\mathrm{sys}}=w_{1}\frac{H_{\mathrm{se}}}{H_{\mathrm{se},\mathrm{sync}}}+w_{2}\frac{S_{\mathrm{th}}}{S_{\mathrm{th},\mathrm{sync}}}$$

where *H*_se,sync_ and *S*_th,sync_ represent the baseline entropy values under conventional synchronous switching. It should be noted that the formulation of this combined entropy metric in (27) assumes that the spectral informational entropy *H*_se_ and the thermodynamic entropy generation *S*th are treated as two independent variables within this specific multi-objective evaluation framework. In addition, *w*_1_ and *w*_2_ are weighting factors (*w*_1_ + *w*_2_ = 1) that depend on how sensitive the specific application is to EMI versus energy efficiency. This flexible allocation of priorities provides a highly adaptable evaluation framework for various industrial environments. In mission-critical applications, such as continuous mining hoists or deep-sea water pumps, preventing electromagnetic interference from affecting communication signals or causing residual current devices to trip prematurely is far more critical than minimizing power consumption. This is because unexpected shutdowns can result in significant economic losses.

To reflect this industrial priority, a higher weight is assigned to spectral order (*w*_1_ = 0.6) than to thermal efficiency (*w*_2_ = 0.4) in this study. The actual experimental numerical values are calculated based on these weights. The baseline metric under synchronous switching is naturally defined as *ψ*_sys_ = 1.0. Under sequential switching, the source-level spectral entropy of the phase voltage decreases from 0.9236 to 0.8665, yielding a spectral entropy ratio of approximately 0.938. Based on the measured active power in Table 4, the thermodynamic entropy generation ratio increases slightly to 1.016 due to the additional RCD snubber losses. Substituting these empirical ratios into (27) yields a combined system entropy of *ψ*_sys_ = 0.969. This calculation proves that although sequential switching slightly increases the normalized *S*th term due to snubber losses, the substantial drop in the normalized *H*_se_ term causes the overall *ψ*_sys_ to decrease.

This combined metric demonstrates quantitatively that the marginal increase in thermodynamic entropy is a worthwhile trade-off for the significant reduction in spectral chaos. This justifies the topology’s application in EMI-sensitive environments. That means a clear engineering trade-off: while the staircase voltage shaping effectively suppresses high-frequency EMI, it introduces additional losses and slightly degrades the output waveform quality. These losses are largely confined to the snubber circuit, leaving the core inverter efficiency virtually unchanged. The approach remains compatible with other EMI mitigation methods and is independent of specific PWM strategies, making it suitable for motor drives across various power ratings and applications.

## 5. Conclusions

This paper introduces a source-level EMI suppression scheme for inverters that uses discrete sequential switching in a series-connected IGBT topology to precisely shape the voltage. Conceptualizing electromagnetic interference as a manifestation of informational disorder, the study shows that optimizing the informational structure of pulse edges can suppress high-frequency emissions. Unlike conventional passive damping methods, which reduce high-frequency noise at the expense of severe heat dissipation, the proposed methodology decouples informational entropy from thermodynamic entropy. It achieves targeted spectral entropy reduction while preserving high IGBT switching speeds and significantly minimizing active-region switching losses. Due to its low control complexity and strong compatibility with existing macroscopic PWM strategies, this approach is highly practical and synergistic for motor drive systems with diverse power ratings. Ultimately, this research bridges the gap between power electronics transient control and information theory, proving that spectral entropy management is a robust tool for holistic EMC optimization.

Future research will focus on two key areas:

First, conducting systematic investigations into parameter sensitivity and multi-objective optimization. The selection of the number of series-connected devices *N* represents a fundamental engineering trade-off: increasing *N* provides higher voltage handling capability and deeper source-level EMI attenuation (lower spectral entropy *H*_se_), but it simultaneously escalates hardware costs, control complexity, and snubber-induced power losses (higher thermodynamic entropy production *S*th.

Second, establishing a comprehensive multi-objective optimization framework will allow for deterministic trade-offs among spectral purity, energy efficiency, and cost across different voltage classes.

## Figures and Tables

**Table 1:** Specifications of the experimental setup.

Name	Parameters
IGBTs	IKW20N60H3
Series-connected IGBTs of each switch leg	4
V_dc_	1500 V
CPLD	EPM240T100C5N
Mine-used flameproof three-phase asynchronous motor	rated power: 3 kW
	rated voltage: 1140 V
	rated current: 2.26 A
	rated speed: 1430 r/min
Carrier frequency	3 kHz
Modulation frequency	50 Hz

**Table 2:** Switching characteristics of IKW20N60H3.

Parameter	Symbol	Value
Turn-on delay time	*t*_d(on)_	48 ns
Rise time	*t*_r_	34 ns
Turn-off delay time	*t*_d(off)_	480 ns
Fall time	*t*_f_	70 ns

**Table 3:** Harmonics information of the staircased voltage and the trapezoidal voltage.

*f*_min_ (*k*, *h*) & *f*_max_ (*k*, *h*)	*f*_min_			*f*_max_			*f*_min_			*f*_max_		
	(1, 1)	(1, 2)	(1, 3)	(1, 1)	(1, 2)	(1, 3)	(2, 1)	(2, 2)	(2, 3)	(2, 1)	(2, 2)	(2, 3)
Frequency (MHz)	0.5	1	1.5	2/3	4/3	2	2.5	3	3.5	5/3	10/3	4
Trapezoidal voltage (dBμV)	121.2	117	112.1	119.4	115.4	111.1	107.1	105.3	102.8	106.3	103.5	101.7
Staircased voltage (dBμV)	93.8	85.1	83.8	104.6	99.9	107.7	80.0	78.2	78.5	91.6	91.1	98.1
Harmonic value difference (dBμV)	27.4	31.9	28.3	14.8	15.5	3.4	27.1	27.1	24.3	14.7	12.4	3.6

**Table 4:** Measured Power Parameters of the three-phase inverter.

Δ*t*	Active Power (W)	Reactive Power (Var)	Apparent Power (VA)
Δ*t* = 0 μs	2933.5	144.6	2941.4
Δ*t* = 0.5 μs	2981.2	170.0	2991.5

## Data Availability

The original contributions presented in this study are included in the article. Further inquiries can be directed to the corresponding author.
